# Genetic analysis of pharmacogenomic VIP variants in the Blang population from Yunnan Province of China

**DOI:** 10.1002/mgg3.574

**Published:** 2019-04-05

**Authors:** Chan Zhang, Weiwei Guo, Yujing Cheng, Qi Li, Xin Yang, Run Dai, Linhao Zhu, Wanlu Chen

**Affiliations:** ^1^ Department of Blood Transfusion The First People’s Hospital of Yunnan Province, The Affiliated Hospital of Kunming University of Science and Technology Kunming, Yunnan China; ^2^ Department of State‐owned Assets Management The First People’s Hospital of Yunnan Province, The Affiliated Hospital of Kunming University of Science and Technology Kunming China; ^3^ Key Laboratory of Molecular Mechanism and Intervention Research for Plateau Diseases of Tibet Autonomous Region, School of Medicine Xizang Minzu University Xianyang, Shaanxi China; ^4^ Key Laboratory of High Altitude Environment and Genes Related to Diseases of Tibet Autonomous Region, School of Medicine Xizang Minzu University Xianyang, Shaanxi China; ^5^ Key Laboratory for Basic Life Science Research of Tibet Autonomous Region, School of Medicine Xizang Minzu University Xianyang, Shaanxi China

**Keywords:** Blang, genetic polymorphism, pharmacogenomics, VIP variants

## Abstract

**Background:**

Genetic polymorphisms in numerous pharmacogenetics studies were regarded as the essential factors involved in the response to or metabolism of drugs. These genetic variants called very important pharmacogenetic (VIP) variants played a role in drugs metabolism, which have been summarized in the PharmGKB database. In this study, we genotyped 80 VIP variants from the PharmGKB in 100 members of Blang volunteers from Yunnan province.

**Methods:**

Based on the PharmGKB database, we genotyped 80 VIP variants loci located in 47 genes. We used χ^2^ tests to evaluate the significant loci between Blang and the other populations, including ASW, CEU, CHB, CHD, GIH, JPT, LWK, MEX, MKK, TSI, and YRI. The global variation distribution of the significant variants was observed from the ALlele FREquency Database. And then, we used *F*‐statistics (Fst), genetic structure, and phylogenetic tree analyses to ascertain the genetic affinity among 12 populations.

**Results:**

Comparing the Blang with the other 11 populations from the HapMap Project, the statistical results revealed that rs3814055 (NC_000003.12:g.119781188C>T) of *nuclear receptor subfamily 1 group I member 2 *(*NR1I2*, OMIM# 603,065) was the most significant variant, followed by rs1540339 (NC_000012.12:g.47863543C>T) of *vitamin D receptor *(*VDR*, OMIM#601,769). Furthermore, we found that genotype frequency of rs3814055 in the Blang was closer to the populations distributed in Miao. And genetic structure and *F*‐statistics indicated that the Blangs had a relatively closer affinity with CHD, CHB, and JPT populations. In addition, the Han nationality in Shaanxi was closer to it.

**Conclusions:**

Our results will complement the pharmacogenomics information of the Blang ethnic group and provide a theoretical basis for safer drug administration for Blang.

## 1 INTRODUCTION

Personalized medicine (Jain, 2009) simply means selection of a best treatment suited for a person on a comprehensive consideration of each patient's characteristics. Its scope is more wider, including pharmacogenetics, pharmacogenomics, and so forth. Pharmacogenomics, a crucial foundation for the development of personalized medicine and patient medication management, enables therapy more precisely.

Furthermore, the Pharmacogenomics Knowledge Base (PharmGKB: http://www.pharmgkb.org) is an extremely useful resource for explaining the gene–drug–disease relationships, more importantly, supporting personalized medicine projects. Recently, a large number of pharmacogenomics studies focused on genetic variations considered to be involved in response to or metabolism of drugs (Evans & McLeod, 2003). These genetic variations also called very important pharmacogenetic (VIP) variants (Peters & McLeod, 2008). At present, there were a total of 246 VIP variants located in 66 genes, which have been summarized in the PharmGKB database.

Numerous studies have elucidated that the importance of ethnicity is great in influencing the frequencies of gene variants. There are 56 ethnic minorities in China, including the Blang ethnic group. The Blang nationality has a population of 91,882 (the fifth national census statistics in 2000), most of whom live in Mount Blang, Xiding, Bada, Mengman, and Daluo areas of Menghai County in Xishuangbanna Dai Autonomous Prefecture of Yunnan province of Southwest China. The others distribute in ***Lincang, Simao, and Baoshan areas (Wang, Hu et al., 2008a). The areas they live in are mild climate and rich products. They are mainly engaged in agricultural production, especially tea planting, which is the origin of the famous Pu'er tea.

This study aims to determine the Blang's genotype and allele frequencies distribution of pharmacogenetic variants. And we compare Blang with the 11 HapMap populations and two national minorities to assess the differences in allele frequencies. The results will complement the database information of pharmacogenomics, better understand the Blang nationality, and provide them with more reasonable individualized health management in the future.

## 2 MATERIALS AND METHODS

### 2.1 Ethical compliance

All participants were informed both in writing and verbally to the procedures and purpose of the study and signed informed consent documents. The study protocol was approved by the Clinical Research Ethics Committee of Xizang Minzu University. It is in accordance with the Department of Health and Human Services (DHHS) regulations for human research subject protection.

### 2.2 Study participants

We randomly recruited about 100 unrelated, healthy Blang people from the Yunnan Province of China. Each participant has undergone rigorous screening criteria. None of the subjects had any diseases including self‐reported cancer history and other diseases. Moreover, despite the influence of the Han and Dai people whose economy and culture development are relatively rapid, they still maintain the characteristics of the nation. They can be seen as representatives of the Blang population.

### 2.3 Variant selection and genotyping

We chose 80 VIP variants loci located in 47 genes from the PharmGKB database. Genomic DNA was extracted from peripheral blood sample using the GoldMag‐Mini Whole Blood Genomic DNA Purification Kit (GoldMag Ltd. Xi'an, China) according to the manufacturer's protocol. NanoDrop 2000C spectrophotometer (Thermo Scientific, Waltham, MA) was used to measure the DNA concentration. We utilized the Sequenom MassARRAY Assay Design 3.0 Software (San Diego, CA) to design Multiplexed SNP MassEXTEND assays (Gabriel, Ziaugra, & Tabbaa, 2009) and genotyped the variants using Sequenom MassARRAY RS1000 (San Diego, CA). Based on the Sequenom Typer 4.0 software (San Diego, CA) used in previous research (He et al., 2015; Jin, Aikemu et al., 2015a; Jin, Yang et al., 2015b; Thomas et al., 2007), we completed data management and analyses.

### 2.4 Statistical analyses

We performed χ^2^ tests and Hardy–Weinberg equilibrium (HWE) analysis by the Microsoft Excel (Redmond, WA) and SPSS 19.0 statistical software platform (SPSS, Chicago, IL). The genotype frequencies of 80 variants in the Blang population were separately compared with those of the other populations, including the Chinese Han in Beijing, China (CHB); the Chinese of metropolitan Denver, Colorado, USA (CHD); the Japanese in Tokyo, Japan (JPT); a residents population in Utah with Northern and Western European Ancestry (CEU); the Gujarati Indians in Houston, Texas, USA (GIH); people with Mexican ancestry living in Los Angeles, California, USA (MEX); the Tuscan people of Italy (TSI); a population of African ancestry in the southwestern USA (ASW); the Luhya people in Webuye, Kenya (LWK); the Maasai people in Kinyawa, Kenya (MKK); and the Yoruba in Ibadan, Nigeria (YRI). All p values of less than 0.05 obtained in this study were two‐sided and Bonferroni's multiple tests were used to calculate the level of significance. After Bonferroni's multiple adjustment, we attempted to discover significantly different sites (*p *< [0.05/(80 × 11)]). Subsequently, we downloaded significant SNP allele frequencies from the ALlele FREquency Database (http://alfred.med.yale.edu, ALFRED) and analyzed the global genetic variation patterns from the HapMap database (Gibbs et al., 2003).

### 2.5 Population genetic structures analysis

In view of the genetic structure of human populations, we used Structure 2.3.4 (Pritchard Lab, Stanford University, USA) (http://pritchardlab.stanford.edu/software/structure_v.2.3.4.html) to observe the variation of the selected VIP variants. On the basis of the Bayesian clustering algorithm approach, we performed structural analysis to assign the samples within a hypothetical *K* number of populations hypothesized by Pritchard, Stephens, and Donnelly (2000). The MCMC analyses for each structure analysis (*K* = 3–10) was run for 10,000 steps after an initial burn‐in period of 10,000 steps. And we used △*K* to calculate to identify the most likely number of clusters by STRUCTURE HARVESTER (Evanno, Regnaut, & Goudet, 2005). Moreover, Wright's *F*‐statistics is the most widely used descriptive statistics in population and evolutionary genetics. (Wright, 1931). We used the program Arlequin version 3.1 to calculate the Fst values to deduce the pairwise distance between populations. Besides, neighbor‐joining method was used to group them in several clusters based on the genetic distance.

## 3 RESULTS

### 3.1 Basic information of the VIP variants

We selected 80 VIP variants from PharmGKB database in 100 members of the Blang population.

The selected single‐nucleotide polymorphisms (SNPs) of PCR primers (listed in Table S1) were designed by the Sequenom MassARRAY Assay Design 3.0 Software. The basic information of the selected variants has been shown in Table 1, including the genes name, their positions, the nucleotide change, the amino acid translation, the allele frequencies, and the genotype frequencies of Blang and the like.

**Table 1:** Basic information of selected VIP variants

SNP	Gene	Full name	Chr	Allele		Position	Amino Acid Translation	Function	Allele A	Allele B	Blang			HWE
				A	B						AA	AB	BB	
rs1045642	ABCB1	ATP binding cassette subfamily B member 1	chr7	A	G	87,138,645	Ile1145Ile	Synonymous	0.335	0.665	12	43	45	0.941
rs1128503	ABCB1	ATP binding cassette subfamily B member 1	chr7	A	G	87,179,601	Gly412Gly	Synonymous	0.590	0.410	36	46	18	0.886
rs2032582	ABCB1	ATP binding cassette subfamily B member 1	chr7	A	C	87,160,618	Ser893Ala	Missense	0.378	0.622	11	43	32	0.841
rs975833	ADH1A	alcohol dehydrogenase 1A (class I), alpha polypeptide	chr4	G	C	100,201,739	—	Intronic	0.365	0.635	11	51	38	0.605
rs1229984	ADH1B	alcohol dehydrogenase 1B (class I), beta polypeptide	chr4	T	C	100,239,319	His48Arg	Missense	0.035	0.965	0	7	93	0.936
rs2066702	ADH1B	alcohol dehydrogenase 1B (class I), beta polypeptide	chr4	G	A	100,229,017	Arg370Cys	Missense	1.000	0.000	100	0	0	—
rs1801253	ADRB1	adrenoceptor beta 1	chr10	G	C	115,805,056	Gly389Arg	Missense	0.350	0.650	14	40	43	0.65
rs1042713	ADRB2	adrenoceptor beta 2	chr5	G	A	148,206,440	Arg16Gly	Missense	0.395	0.605	10	59	31	0.064
rs1042714	ADRB2	adrenoceptor beta 2	chr5	G	C	148,206,473	Gln27Glu	Missense	0.050	0.950	0	10	90	0.87
rs1800888	ADRB2	adrenoceptor beta 2	chr5	C	T	148,206,885	Thr164Ile	Missense	1.000	0.000	100	0	0	—
rs2066853	AHR	aryl hydrocarbon receptor	chr7	G	A	17,379,110	Arg554Lys	Missense	0.845	0.155	73	23	4	0.475
rs6151031	ALDH1A1	aldehyde dehydrogenase 1 family member A1	chr9	—	CTGGTGAGG AGAGAACC	72,953,467	—	—	0.953	0.047	87	9	0	0.89
rs1800497	ANKK1	ankyrin repeat and kinase domain containing 1	chr11	G	A	113,270,828	Glu713Lys	Missense	0.720	0.280	54	36	10	0.563
rs4680	COMT	catechol‐O‐methyltransferase	chr22	G	A	19,951,271	Val158Met	Missense	0.860	0.140	72	28	0	0.266
rs1801272	CYP2A6	cytochrome P450 family 2 subfamily A member 6	chr19	A	T	41,354,533	Leu160His	Missense	0.000	1.000	0	0	100	—
rs28399433	CYP2A6	cytochrome P450 family 2 subfamily A member 6	chr19	G	T	41,356,379	—	—	0.200	0.800	4	32	64	1
rs28399444	CYP2A6	cytochrome P450 family 2 subfamily A member 6	chr19	G	A	41,354,190	—	Frameshift	0.000	1.000	0	0	100	—
rs28399454	CYP2A6	cytochrome P450 family 2 subfamily A member 6	chr19	C	T	41,351,267	Val365Met	Missense	1.000	0.000	100	0	0	—
rs28399499	CYP2B6	cytochrome P450 family 2 subfamily B member 6	chr19	T	C	41,518,221	Ile328Thr	Missense	1.000	0.000	100	0	0	—
rs3745274	CYP2B6	cytochrome P450 family 2 subfamily B member 6	chr19	G	T	41,512,841	Gln172His	Missense	0.485	0.515	21	55	24	0.601
rs4986893	CYP2C19	cytochrome P450 family 2 subfamily C member 19	chr10	A	G	96,540,410	Trp212null	Stop Codon	0.025	0.975	0	5	95	0.968
rs1799853	CYP2C9	cytochrome P450 family 2 subfamily C member 9	chr10	C	T	96,702,047	Arg144Cys	Missense	1.000	0.000	100	0	0	—
rs16947	CYP2D6	cytochrome P450 family 2 subfamily D member 6	chr22	A	G	42,523,943	Arg296Cys	Missense	0.210	0.790	0	42	58	0.029
rs28371706	CYP2D6	cytochrome P450 family 2 subfamily D member 6	chr22	G	A	42,525,772	Thr107Ile	Missense	1.000	0.000	100	0	0	—
rs28371725	CYP2D6	cytochrome P450 family 2 subfamily D member 6	chr22	A	G	42,523,805	—	Intronic	0.130	0.870	1	24	75	0.83
rs5030656	CYP2D6	cytochrome P450 family 2 subfamily D member 6	chr22	—	AAG	42,128,174	deletes K281	Non‐synonymous	0.000	1.000	0	0	100	—
rs59421388	CYP2D6	cytochrome P450 family 2 subfamily D member 6	chr22	C	T	42,523,610	Val338Met	Missense	1.000	0.000	100	0	0	—
rs61736512	CYP2D6	cytochrome P450 family 2 subfamily D member 6	chr22	C	T	42,525,134	Val136Met	Missense	1.000	0.000	100	0	0	—
rs12721634	CYP3A4	cytochrome P450 family 3 subfamily A member 4	chr7	C	T	99,381,661	Leu15Pro	Missense	0.000	1.000	0	0	100	—
rs2740574	CYP3A4	cytochrome P450 family 3 subfamily A member 4	chr7	A	G	99,382,096	—	—	1.000	0.000	100	0	0	—
rs4986909	CYP3A4	cytochrome P450 family 3 subfamily A member 4	chr7	G	A	99,359,670	Pro415Leu	Missense	1.000	0.000	100	0	0	—
rs4986910	CYP3A4	cytochrome P450 family 3 subfamily A member 4	chr7	A	G	99,358,524	Met444Thr	Missense	1.000	0.000	100	0	0	—
rs4986913	CYP3A4	cytochrome P450 family 3 subfamily A member 4	chr7	G	A	99,358,459	Pro466Ser	Missense	1.000	0.000	100	0	0	—
rs10264272	CYP3A5	cytochrome P450 family 3 subfamily A member 5	chr7	C	T	99,262,835	Lys208Lys	Synonymous	1.000	0.000	100	0	0	—
rs3918290	DPYD	dihydropyrimidine dehydrogenase	chr1	C	T	97,915,614	—	Splice acceptor	1.000	0.000	100	0	0	—
rs6277	DRD2	dopamine receptor D2	chr11	G	A	113,283,459	Pro319Pro	Synonymous	0.975	0.025	95	5	0	0.968
rs1138272	GSTP1	glutathione S‐transferase pi 1	chr11	C	T	67,353,579	Ala114Val	Missense	1.000	0.000	100	0	0	—
rs1695	GSTP1	glutathione S‐transferase pi 1	chr11	A	G	67,352,689	Ile105Val	Missense	0.740	0.260	55	38	7	0.992
rs17238540	HMGCR	3‐hydroxy−3‐methylglutaryl‐CoA reductase	chr5	G	T	74,655,498	—	Splice acceptor	0.000	1.000	0	0	100	—
rs17244841	HMGCR	3‐hydroxy−3‐methylglutaryl‐CoA reductase	chr5	A	T	74,642,855	—	Intronic	0.929	0.071	87	8	3	0.001
rs3846662	HMGCR	3‐hydroxy−3‐methylglutaryl‐CoA reductase	chr5	A	G	74,651,084	—	Intronic	0.465	0.535	22	48	29	0.968
rs12720441	KCNH2	potassium voltage‐gated channel subfamily H member 2	chr7	G	A	150,647,304	Arg784Trp	Missense	1.000	0.000	100	0	0	—
rs36210421	KCNH2	potassium voltage‐gated channel subfamily H member 2	chr7	G	T	150,644,428	Arg1047Leu	Missense	1.000	0.000	99	0	0	—
rs3807375	KCNH2	potassium voltage‐gated channel subfamily H member 2	chr7	C	T	150,667,210	—	Intronic	0.200	0.800	6	28	66	0.458
rs1801131	MTHFR	methylenetetrahydrofolate reductase	chr1	T	G	11,854,476	Glu429Ala	Missense	0.795	0.205	65	29	6	0.544
rs1801133	MTHFR	methylenetetrahydrofolate reductase	chr1	G	A	11,856,378	Ala222Val	Missense	0.770	0.230	62	30	8	0.31
rs1800566	NQO1	NAD(P)H quinone dehydrogenase 1	chr16	G	A	69,711,242	Pro187Ser	Missense	0.595	0.405	32	55	13	0.369
rs3814055	NR1I2	nuclear receptor subfamily 1 group I member 2	chr3	C	T	119,500,035	—	5'‐UTR	0.940	0.060	88	12	0	0.816
rs1065776	P2RY1	purinergic receptor P2Y1	chr3	C	T	152,553,628	Ala19Ala	Synonymous	0.875	0.125	64	19	1	0.953
rs701265	P2RY1	purinergic receptor P2Y1	chr3	A	G	152,554,357	Val262Val	Synonymous	0.695	0.305	45	49	6	0.297
rs2046934	P2RY12	purinergic receptor P2Y12	chr3	G	A	151,057,642	—	Intronic	0.085	0.915	0	17	83	0.65
rs5629	PTGIS	prostaglandin I2 synthase	chr20	G	T	48,129,706	Arg373Arg	Synonymous	0.885	0.115	77	23	0	0.43
rs689466	PTGS2	prostaglandin‐endoperoxide synthase 2	chr1	T	C	186,650,751	—	—	0.596	0.404	36	46	17	0.941
rs1805124	SCN5A	sodium voltage‐gated channel alpha subunit 5	chr3	T	C	38,645,420	His558Arg	Missense	0.890	0.110	81	16	3	0.188
rs6791924	SCN5A	sodium voltage‐gated channel alpha subunit 5	chr3	G	A	38,674,699	Arg34Cys	Missense	1.000	0.000	100	0	0	—
rs7626962	SCN5A	sodium voltage‐gated channel alpha subunit 5	chr3	T	G	38,620,907	Ser1103Tyr	Missense	0.000	1.000	0	0	100	—
rs1051266	SLC19A1	solute carrier family 19 member 1	chr21	T	C	46,957,794	His27Arg	Missense	0.436	0.564	13	56	25	0.123
rs12659	SLC19A1	solute carrier family 19 member 1	chr21	C	T	46,951,556	Pro232Pro	Synonymous	0.556	0.444	25	59	14	0.094
rs4149056	SLCO1B1	solute carrier organic anion transporter family member 1B1	chr12	T	C	21,331,549	Val174Ala	Missense	0.965	0.035	93	7	0	0.936
rs1801030	SULT1A1	sulfotransferase family 1A member 1	chr16	C	T	28,617,485	Val 223Met	Missense	0.000	1.000	0	0	100	—
rs3760091	SULT1A1	sulfotransferase family 1A member 1	chr16	G	C	28,609,479	—	Intronic	0.355	0.645	6	59	35	0.016
rs1142345	TPMT	thiopurine S‐methyltransferase	chr6	T	C	18,130,918	Tyr240Cys	Missense	0.985	0.015	95	3	0	0.988
rs1800460	TPMT	thiopurine S‐methyltransferase	chr6	A	G	18,139,228	Ala154Thr	Missense	0.000	1.000	0	0	100	—
rs1800462	TPMT	thiopurine S‐methyltransferase	chr6	C	G	18,143,955	Ala80Pro	Missense	0.000	1.000	0	0	98	—
rs34489327	TS	thymidylate synthetase	chr18	Del			—	3'‐UTR	1.000	0.000	100	0	0	—
rs10929302	UGT1A1	UDP glucuronosyltransferase family	chr2	G	A	234,665,782	—	Intronic	0.880	0.120	78	20	2	0.869
rs4124874	UGT1A1	UDP glucuronosyltransferase family 1 member A1	chr2	T	G	234,665,659	—	Intronic	0.530	0.470	32	42	26	0.292
rs4148323	UGT1A1	UDP glucuronosyltransferase family 1 member A1	chr2	G	A	234,669,144	Gly71Arg	Missense	0.845	0.155	71	27	2	0.954
rs10735810	VDR	vitamin D (1,25‐dihydroxyvitamin D3) receptor	chr12	A	G	48,272,895	Met1Thr	Missense	0.571	0.429	28	57	14	0.22
rs11568820	VDR	vitamin D (1,25‐dihydroxyvitamin D3) receptor	chr12	C	T	48,302,545	—	—	0.196	0.804	4	21	49	0.694
rs1540339	VDR	vitamin D (1,25‐dihydroxyvitamin D3) receptor	chr12	C	T	48,257,326	—	Intronic	0.340	0.660	13	42	45	0.814
rs1544410	VDR	vitamin D (1,25‐dihydroxyvitamin D3) receptor	chr12	C	T	48,239,835	—	Intronic	0.975	0.025	94	5	0	0.967
rs2228570	VDR	vitamin D (1,25‐dihydroxyvitamin D3) receptor	chr12	T	C	48,272,895	Met1Thr	Missense	0.575	0.425	29	57	14	0.251
rs2239179	VDR	vitamin D (1,25‐dihydroxyvitamin D3) receptor	chr12	T	C	48,257,766	—	Intronic	0.000	0.000	0	0	0	—
rs2239185	VDR	vitamin D (1,25‐dihydroxyvitamin D3) receptor	chr12	G	A	48,244,559	—	Intronic	0.695	0.305	43	53	4	0.044
rs731236	VDR	vitamin D (1,25‐dihydroxyvitamin D3) receptor	chr12	A	G	48,238,757	Ile352Ile	Synonymous	0.975	0.025	95	5	0	0.968
rs7975232	VDR	vitamin D (1,25‐dihydroxyvitamin D3) receptor	chr12	C	A	48,238,837	—	Intronic	0.695	0.305	43	53	4	0.044
rs7294	VKORC1	vitamin K epoxide reductase complex subunit 1	chr16	C	T	31,102,321	—	3'‐UTR	0.874	0.126	75	23	1	0.87
rs9923231	VKORC1	vitamin K epoxide reductase complex subunit 1	chr16	A	C	31,096,368	—	—	1.000	0.000	100	0	0	—
rs9934438	VKORC1	vitamin K epoxide reductase complex subunit 1	chr16	G	A	31,104,878	—	Intronic	0.125	0.875	1	23	76	0.876

Notes

SNP: single‐nucleotide polymorphism; HWE: Hardy–Weinberg equilibrium. The GenBank reference of the above genes were as follows: *ABCB1* (NC_000007.14), *ADH1A* (NC_000004.12), *ADH1B *(NC_000004.12), *ADRB1* (NC_000010.11), *ADRB2* (NC_000005.10), *AHR* (NC_000007.14), *ALDH1A1* (NC_000009.12), *ANKK1* (NC_000011.10), *COMT* (NC_000022.11), *CYP2A6* (NC_000019.10), *CYP2B6* (NC_000019.10), *CYP2C19* (NC_000010.11), *CYP2C9* (NC_000010.11), *CYP2D6* (NC_000022.11), *CYP3A4* (NC_000007.14), *CYP3A5* (NC_000007.14), *DPYD* (NC_000001.11), *DRD2* (NC_000011.10), *GSTP1* (NC_000011.10), *HMGCR* (NC_000005.10), *KCNH2* (NC_000007.14), *MTHFR* (NC_000001.11), *NQO1* (NC_000016.10), *NR1I2* (NC_000003.12), *P2RY1* (NC_000003.12), *P2RY12* (NC_000003.12), *PTGIS* (NC_000020.11), *PTGS2* (NC_000001.11), *SCN5A* (NC_000003.12), *SLC19A1* (NC_000021.9), *SLCO1B1* (NC_000012.12), *SULT1A1* (NC_000016.10), *TPMT* (NC_000006.12), *TS* (NC_000018.10), *UGT1A1* (NC_000002.12), *VDR* (NC_000012.12), *VKORC1* (NC_000016.10).

### 3.2 Analyses of 80 loci among 12 populations

The average variants call rate of the results was over 95%. All selected loci meet the HWE. Using chi‐square test, we compared the Blangs and the 11 populations of the genotype frequencies distribution of 80 loci. Before adjustment (*p* < 0.05), we found that some loci were different (not shown). When compared to the 11 groups (ASW, CEU, CHB, CHD, GIH, JPT, LWK, MEX, MKK, TSI, and YRI) and Blang without adjustment, the number of significantly different variants in the Blang population was 23, 30, 17, 30, 30, 21, 26, 21, 25, 22, and 35, respectively (data no shown). After adjustment (*p* < [0.05/(80 × 11)], listed in Table 2), there were 15, 20, 6, 25, 25, 7, 19, 7, 20, 15, and 26 loci of significant differences between Blang and the 11 populations, respectively. While there were contrasts in the two sets of data, there were also similarities. It was also noteworthy that the different loci between CHB and the Blang were the least.

**Table 2:** Significant VIP variants in the Blangs compared with the 11 populations after Bonferroni's multiple adjustment

SNP ID	Gene	*p* < 0.05/（80*11）										
		ASW	CEU	CHB	CHD	GIH	JPT	LWK	MEX	MKK	TSI	YRI
rs1045642	ABCB1	0.059	**2.873E−06**	0.277	0.024	0.292	0.022	—	0.076	**2.808E−05**	0.042	**4.723E−07**
rs1128503	ABCB1	**2.072E−09**	0.005	0.072	—	**2.890E−10**	0.974	**2.319E−17**	0.070	**5.876E−19**	0.009	**7.463E−19**
rs2032582	ABCB1	**1.486E−06**	0.161	0.001	—	—	0.003	**5.000E−16**	0.668	**2.232E−12**	0.465	—
rs975833	ADH1A	—	**3.544E−08**	0.001	—	—	0.011	—	—	—	—	**9.068E−09**
rs1229984	ADH1B	—	—	**3.393E−25**	—	—	**9.317E−25**	—	—	—	—	—
rs2066702	ADH1B	**1.065E−10**	—	—	**1.056E−19**	**2.444E−05**	—	**4.081E−07**	—	—	—	**5.646E−15**
rs1801253	ADRB1	—	0.785	0.217	—	—	0.004	—	—	—	—	0.365
rs1042713	ADRB2	0.258	**4.559E−07**	0.481	—	—	0.001	0.028	0.011	0.097	**1.166E−07**	0.003
rs1042714	ADRB2	—	**1.530E−12**	—	**8.438E−14**	**9.243E−23**	0.305	—	—	—	—	0.002
rs1800888	ADRB2	—	—	—	—	—	—	—	—	—	—	—
rs2066853	AHR	**1.696E−05**	0.181	**1.202 E−06**	—	—	**5.580E−09**	**1.516E−10**	0.358	**2.034E−06**	0.085	**5.504E−09**
rs6151031	ALDH1A1	—	—	—	—	—	—	—	—	—	—	—
rs1800497	ANKK1	0.107	0.144	0.025	0.000	0.077	0.031	0.129	0.024	0.115	0.261	0.012
rs4680	COMT	0.002	**2.205E−11**	0.000	—	—	0.001	0.000	**2.862E−06**	0.001	**2.593E−10**	0.000
rs1801272	CYP2A6	—	**1.805E−35**	—	**6.726E−41**	**7.698E−40**	**5.380E−32**	—	—	—	—	—
rs28399433	CYP2A6	—	—	—	—	—	—	—	—	—	—	—
rs28399444	CYP2A6	—	—	—	—	—	—	—	—	—	—	—
rs28399454	CYP2A6	—	—	—	—	—	—	—	—	—	—	—
rs28399499	CYP2B6	0.000	—	—	—	—	—	—	—	0.168	—	**2.037E−06**
rs3745274	CYP2B6	0.000	**1.314E−06**	**1.166E−10**	—	—	**2.002E−10**	0.000	0.001	0.007	**2.326E−05**	0.100
rs4986893	CYP2C19	—	—	—	—	—	—	—	—	—	—	—
rs1799853	CYP2C9	—	—	—	—	—	—	—	—	—	—	—
rs16947	CYP2D6	—	—	—	0.248	0.003	—	—	—	—	—	—
rs28371706	CYP2D6	—	—	—	—	—	—	—	—	—	—	—
rs28371725	CYP2D6	—	—	—	—	—	—	—	—	—	—	—
rs5030656	CYP2D6	—	—	—	**2.373E−13**	**7.203E−13**	—	—	—	—	—	—
rs59421388	CYP2D6	—	—	—	—	—	—	—	—	—	—	—
rs61736512	CYP2D6	—	—	—	—	—	—	—	—	—	—	—
rs12721634	CYP3A4	—	—	—	**9.801E−37**	**3.439E−16**	—	—	—	—	—	—
rs2740574	CYP3A4	—	—	—	—	—	—	—	—	—	—	—
rs4986909	CYP3A4	—	—	—	—	—	—	—	—	—	—	—
rs4986910	CYP3A4	—	—	—	—	—	—	—	—	—	—	—
rs4986913	CYP3A4	—	—	—	—	—	—	—	—	—	—	—
rs10264272	CYP3A5	—	—	—	**7.700E−31**	**2.008E−21**	—	**1.445E−12**	—	**8.823E−08**	—	**8.948E−09**
rs3918290	DPYD	—	—	—	**3.132E−18**	**1.273E−33**	—	—	—	—	—	—
rs6277	DRD2	—	**1.663E−22**	—	—	—	—	—	—	—	—	—
rs1138272	GSTP1	—	—	—	—	—	—	—	0.001	—	—	—
rs1695	GSTP1	0.003	0.003	0.231	—	—	0.000	**1.935E−06**	**1.304E−05**	0.079	0.514	0.014
rs17238540	HMGCR	—	—	—	—	—	—	—	—	—	—	—
rs17244841	HMGCR	—	—	—	—	—	—	—	—	—	—	—
rs3846662	HMGCR	**1.111E−07**	0.084	0.994	—	**1.222E−23**	0.607	**3.482E−18**	0.030	**2.452E−11**	0.257	**8.429E−20**
rs12720441	KCNH2	—	—	—	—	—	—	—	—	—	—	—
rs36210421	KCNH2	—	—	—	**2.249E−07**	**2.963E−20**	—	—	—	—	—	—
rs3807375	KCNH2	0.042	**5.814E−16**	0.172	**3.093E−18**	**1.580E−11**	0.165	0.619	0.001	0.048231	**1.349E−15**	0.627
rs1801131	MTHFR	0.458	0.006	0.439	0.013	0.448	0.440	0.688	0.753	0.308	0.066	0.035
rs1801133	MTHFR	0.013	0.076	**1.559E−05**	—	—	0.009	0.002	0.002	0.000	0.000	0.001
rs1800566	NQO1	0.001	**2.084E−06**	0.135	—	—	0.403	**3.384E−06**	0.218	**3.058E−08**	0.000	**4.510E−06**
rs3814055	NR1I2	**1.029E−07**	**9.604E−11**	**2.269E−07**	**1.593E−19**	**1.270E−25**	**1.087E−06**	**1.235E−07**	**1.475E−07**	**3.575E−06**	**8.035E−13**	**1.283E−07**
rs1065776	P2RY1	—	—	—	**2.296E−11**	**2.388E−19**	—	—	—	—	—	—
rs701265	P2RY1	**1.620E−09**	0.007	0.293	**5.222E−08**	**3.034E−13**	0.266	**6.247E−19**	0.052	**8.827E−19**	0.001	**1.022E−20**
rs2046934	P2RY12	—	0.001	0.010	—	—	0.020	—	—	—	—	0.004
rs5629	PTGIS	0.124	0.006	0.003	—	—	0.008	—	0.001	0.701	**9.651E−07**	0.408
rs689466	PTGS2	**1.039E−06**	**1.169E−06**	0.175	0.007	0.001	0.339	**4.422E−15**	0.029	**5.0037E−21**	**1.203E−05**	**1.306E−11**
rs1805124	SCN5A	0.003	0.008	0.194	**1.046E−15**	**1.110E−07**	0.082	0.000	0.334	**2.198E−08**	0.004	**1.258E−06**
rs6791924	SCN5A	—	—	—	**2.606E−22**	**1.626E−07**	—	—	—	—	—	—
rs7626962	SCN5A	—	—	—	**4.880E−15**	**7.055E−29**	—	—	—	—	—	0.001
rs1051266	SLC19A1	0.531	0.818	0.056	—	—	0.018	**8.244E−09**	0.059	**9.767E−13**	0.181	**1.011E−07**
rs12659	SLC19A1	—	—	—	—	—	—	—	—	—	—	—
rs4149056	SLCO1B1	0.382	0.000	0.000	**4.768E−12**	**3.391E−15**	0.024	—	—	0.007	**4.259E−07**	—
rs1801030	SULT1A1	—	—	—	**5.982E−36**	**3.565E−30**	—	—	—	—	—	—
rs3760091	SULT1A1	—	—	—	**1.349E−12**	0.255	—	—	—	—	—	—
rs1142345	TPMT	—	—	—	**1.031E−15**	**8.286E−19**	—	0.001	0.034	—	—	0.269
rs1800460	TPMT	—	—	—	—	—	—	—	—	—	—	—
rs1800462	TPMT	—	—	—	**2.714E−22**	**1.166E−12**	—	—	—	—	—	—
rs34489327	TS	—	—	—	—	—	—	—	—	—	—	—
rs10929302	UGT1A1	—	0.002	0.592	**2.608E−05**	**4.574E−09**	0.610	—	—	—	—	**1.298E−05**
rs4124874	UGT1A1	**8.170E−06**	0.630	0.0002	**2.226E−07**	**3.725E−18**	0.029	**1.136E−13**	0.832	**1.947E−13**	0.277	**1.265E−17**
rs4148323	UGT1A1	—	**2.428E−05**	0.108	0.536	0.000	0.769	—	0.007	—	—	**2.428E−05**
rs10735810	VDR	**1.259E−10**	0.001	0.003	—	—	**4.321E−07**	**1.916E−15**	0.299	**3.465E−15**	0.000	**2.414E−14**
rs11568820	VDR	0.133	**2.969E−22**	**4.962E−08**	—	—	**1.086E−07**	0.061	**8.376E−15**	0.618	**7.171E−18**	**5.433E−07**
rs1540339	VDR	**1.172E−09**	**1.135E−07**	0.520	**6.552E−26**	**1.452E−05**	0.284	**4.490E−19**	0.000	**2.537E−20**	**1.931E−07**	**1.484E−16**
rs1544410	VDR	—	**9.321E−19**	—	—	—	—	**1.521E−09**	**3.563E−08**	**1.478E−16**	**2.207E−17**	**1.580E−11**
rs2228570	VDR	—	—	—	**1.814E−11**	0.110	—	—	—	—	—	—
rs2239179	VDR	—	—	—	—	—	—	—	—	—	—	—
rs2239185	VDR	—	—	0.027	—	—	0.161	—	—	—	—	**8.001E−06**
rs731236	VDR	**3.289E−08**	**6.439E−19**	—	—	—	—	**1.059E−09**	**7.603E−09**	**4.993E−24**	**1.560E−17**	**1.221E−12**
rs7975232	VDR	**1.511E−08**	**1.887E−08**	0.040	—	—	0.127	**1.767E−15**	0.016	**1.807E−14**	**1.129E−08**	**9.107E−11**
rs7294	VKORC1	**4.496E−11**	**2.436E−07**	0.051	0.014	0.000	0.553	**1.328E−10**	0.000	**3.526E−14**	**4.204E−06**	**9.144E−15**
rs9923231	VKORC1	—	—	—	**1.109E−40**	**1.328E−10**	—	—	—	—	—	—
rs9934438	VKORC1	**1.112E−25**	**6.971E−18**	0.055	—	—	0.551	**1.212E−34**	**1.623E−11**	**6.354E−37**	**9.903E−14**	**1.557E−42**

Notes

SNP: single‐nucleotide polymorphism; HWE: Hardy–Weinberg equilibrium. ASW, a population of African ancestry in the southwestern USA; CEU, a residents population in Utah with Northern and Western European Ancestry; CHB, the Chinese Han in Beijing, China; CHD, the population of metropolitan Denver, Colorado, USA; GIH, the Gujarati Indians in Houston, Texas, USA; JPT, the Japanese population in Tokyo, Japan; LWK, the Chinese living in Luhya in Webuye, Kenya; MEX, people with Mexican ancestry living in Los Angeles, California, USA; MKK, the Maasai people in Kinyawa, Kenya; TSI, the Tuscan people of Italy; YRI, the Yoruba in Ibadan, Nigeria. The GenBank reference of the above genes were as follows: *ABCB1* (NC_000007.14), *ADH1A* (NC_000004.12), *ADH1B *(NC_000004.12), *ADRB1* (NC_000010.11), *ADRB2* (NC_000005.10), *AHR* (NC_000007.14), *ALDH1A1* (NC_000009.12), *ANKK1* (NC_000011.10), *COMT* (NC_000022.11), *CYP2A6* (NC_000019.10), *CYP2B6* (NC_000019.10), *CYP2C19* (NC_000010.11), *CYP2C9* (NC_000010.11), *CYP2D6* (NC_000022.11), *CYP3A4* (NC_000007.14), *CYP3A5* (NC_000007.14), *DPYD* (NC_000001.11), *DRD2* (NC_000011.10), *GSTP1* (NC_000011.10), *HMGCR* (NC_000005.10), *KCNH2* (NC_000007.14), *MTHFR* (NC_000001.11), *NQO1* (NC_000016.10), *NR1I2* (NC_000003.12), *P2RY1* (NC_000003.12), *P2RY12* (NC_000003.12), *PTGIS* (NC_000020.11), *PTGS2* (NC_000001.11), *SCN5A* (NC_000003.12), *SLC19A1* (NC_000021.9), *SLCO1B1* (NC_000012.12), *SULT1A1* (NC_000016.10), *TPMT* (NC_000006.12), *TS* (NC_000018.10), *UGT1A1* (NC_000002.12), *VDR* (NC_000012.12), *VKORC1* (NC_000016.10).

Bold type indicates that the locus has statistically significant.

However, through a comparison of before and after adjustment, the distribution of rs1801133 (HGVS: NM_001330358.1:c.788C>T) and rs4680 (HGVS: NM_000754.3:c.472G>A) in populations has changed. After correction for multiple tests, rs1801133 became less significant in ASW, JPT, LWK, MEX, MKK, TSI, YRI, except CHB. Besides, rs4680 were detected significant differences between CEU, MEX, TSI, and Blang. In the populations of ASW, CHB, JPT, LWK, MKK, and YRI, its differences disappeared. Nonetheless, some variants varied little, not even a bit, such as rs11568820, rs1544410, and so forth.

After analysis of Table 2, significant variants in some genes were distributed in every population, such as *VDR* and *NR1I2*. There were rs10735810, rs11568820, rs1540339, rs1544410, rs2228570, rs2239179, rs2239185, rs731236, and rs7975232 distributed in *VDR *(*vitamin D receptor*), which encodes the nuclear hormone receptor for vitamin D3. Although failing to make amino acid changed, rs1540339 was also very significant among the nine populations except CHB, JPT, and MEX. Although rs2228570 (HGVS: NM_000376.2:c.2 T>G) was, the only one SNP changing amino acid, located in exon 2 of *VDR*, it was still prominent in the CHD.

**Figure 1:**
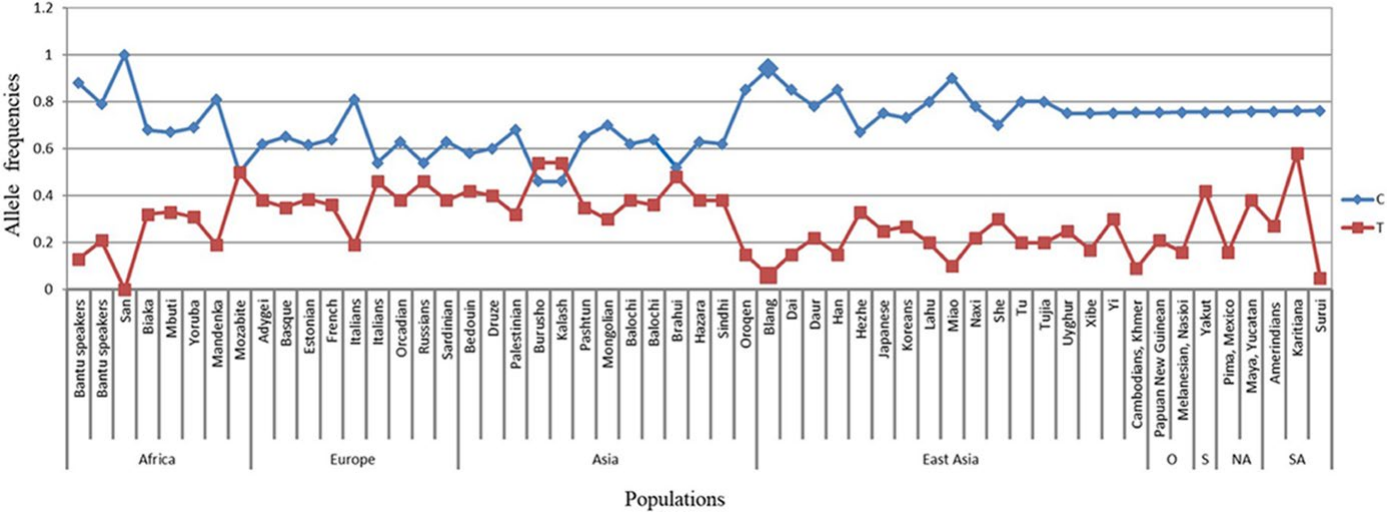
The frequencies of rs3814055 in the different populations. NA, North America; SA, South America; S, Siberia; O, Oceania

**Figure 2:**
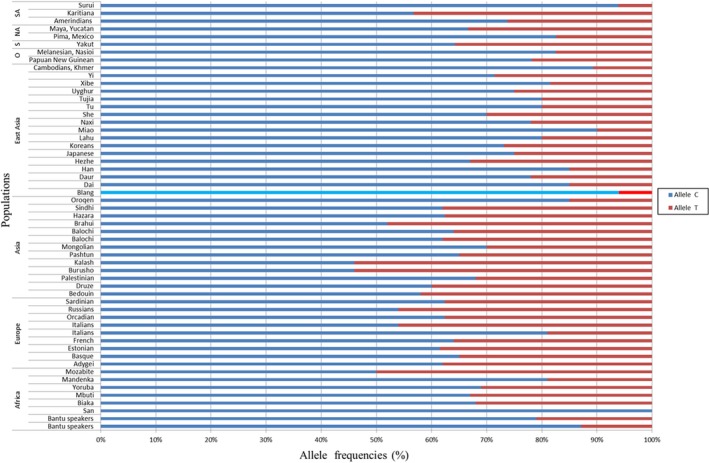
Rs3814055 frequencies in different populations of the world. NA, North America; SA, South America; S, Siberia; O, Oceania

Although rs3814055 in *NR1I2 *changed little, significant differences still existed. We downloaded the associated data of rs3814055 from the website (http://alfred.med.yale.edu). As seen from the Figures 1 and 2, the frequency of the Blangs was closer to the populations distributed in East Asia, especially Miao. On the whole, the frequencies of the allele C of rs3814055, ranged from 67% to 94%, were higher in East Asia than the other populations. The Blang population was the highest among them, so attention should be paid to its allele C.

### 3.3 The relationship between 23 populations

**Figure 3:**
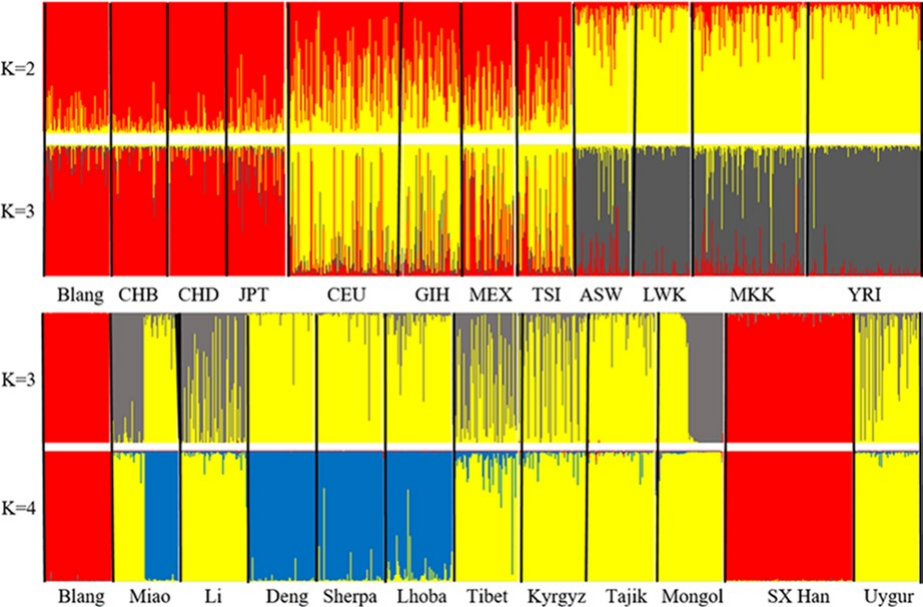
Analysis the genetic structure between Blang and the 23 populations. *K* denotes the possible numbers of parental population clusters. Each vertical bar represents a person, dividing into color sections. *K* = 2, 3 were used to assess the genetic relationship between Blang and 11 global populations. And the genetic relationship between 11 ethnic groups from China and Blang were evaluated by *K* = 3, 4. ASW: ASW: a population of African ancestry in southwestern USA; CEU: a residents population in Utah with Northern and Western European Ancestry; CHB: the Chinese Han in Beijing, China; CHD: Chinese in Metropolitan Denver, Colorado, USA; GIH: Gujarati Indians in Houston, Texas, USA; JPT: Japanese in Tokyo, Japan; LWK: Luhya people in Webuye, Kenya; MEX: people with Mexican ancestry in Los Angeles, California, USA; MKK: Maasai people in Kinyawa, Kenya; TSI: Toscans in Italy; YRI: Yoruba in Ibadan, Nigeria; SX Han, Shaanxi Han. A: Comparing the Blangs with the other 11 populations from the International HapMap Project, Blang was closer to CHB, CHD, and JPT. B: The Han nationality in Shaanxi was very close to the Blangs within China

We used Structure 2.3.1 Software to analyze the genetic structure of the 23 populations in order to further identify the relationships between them throughout the world. Different *K* values ranging from 2 to 10 were hypothetically in structure analysis. And, the results of *K* = 2,3 among global populations and the results of *K* = 3,4 ethnic groups from China were shown in Figure 3. The cluster analysis indicated that when *K* = 3, the group was divided into three subgroups (subgroups 1: Blang, CHB, CHD, JPT, SX Han; subgroups 2: CEU, GIH, MEX, TSI, Deng, Sherpa, Lhoba, Kyrgyz, Tajik, Uygur; subgroups 3: ASW, LWK, MKK, YRI, Miao, Li, Tibet, Mongol) based on relative majority of likelihood to assign individuals to subgroups. The results illustrated that Blang had a relatively closer affinity with CHB, CHD, and JPT. In accordance with the Table 2, the results were confirmed. Likewise, when comparing ethnic groups within China, we found that Blang was closer to SX Han.

**Figure 4:**
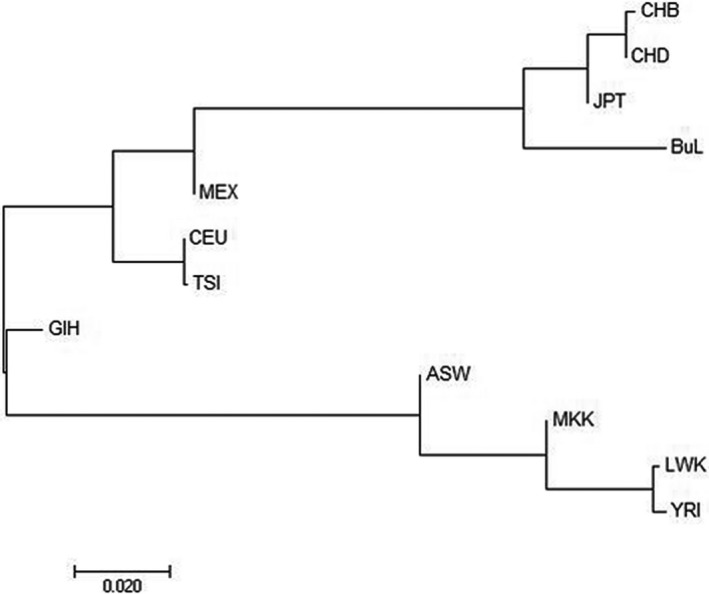
The phylogenetic tree was reconstructed by the neighboring‐joining method among 12 populations

Based on genetic structure, we further assessed the genetic relationship among 12 populations by using pairwise Fst values (Table 3). As mentioned in it, it was clear that the differences between CHB, CHD, JPT, and Blang (Fst = 0.04728, 0.04259, and 0.04914, respectively) were smaller. The smaller the Fst value, the more similar they were. The results indicated that the Blang and the other three groups had a relatively closer affinity, followed by MEX. As presented by the phylogenetic tree (Figure 4) about 12 populations in the same Fst‐based way, the results were verified again.

**Table 3:** Fst values among 12 populations

	BuL	CHB	CHD	JPT	CEU	GIH	MEX	TSI	ASW	LWK	MKK	YRI
BuL	0											
CHB	**0.04728**	0										
CHD	**0.04259**	−0.00161	0									
JPT	**0.04914**	0.00586	0.00761	0								
CEU	0.14462	0.13026	0.12708	0.11499	0							
GIH	0.15465	0.15697	0.15321	0.14338	0.03311	0						
MEX	**0.09721**	0.08424	0.07821	0.08033	0.02248	0.05258	0					
TSI	0.14058	0.11524	0.11626	0.10172	0.00012	0.04047	0.02447	0				
ASW	0.17273	0.1955	0.19394	0.17125	0.12124	0.08173	0.11144	0.12461	0			
LWK	0.23967	0.26654	0.26764	0.23703	0.18539	0.14618	0.18563	0.19061	0.01719	0		
MKK	0.20378	0.23189	0.23406	0.19985	0.13638	0.10553	0.15181	0.14253	0.01888	0.01336	0	
YRI	0.23439	0.26827	0.27045	0.23703	0.19138	0.14351	0.19235	0.1978	0.01513	0.00383	0.01359	0

Notes

ASW, a population of African ancestry in the southwestern USA; CEU, a residents population in Utah with Northern and Western European Ancestry; CHB, the Chinese Han in Beijing, China; CHD, the population of metropolitan Denver, Colorado, USA; GIH, the Gujarati Indians in Houston, Texas, USA; JPT, the Japanese population in Tokyo, Japan; LWK, the Chinese living in Luhya in Webuye, Kenya; MEX, people with Mexican ancestry living in Los Angeles, California, USA; MKK, the Maasai people in Kinyawa, Kenya; TSI, the Tuscan people of Italy; YRI, the Yoruba in Ibadan, Nigeria.

## 4 DISCUSSION

There is increasing interested in personalized medicine, because of genetic variations leading to each person's different metabolism of and reactions to some drugs. In our results, we genotyped the pharmacogenomic VIP variants in the Blang population. The conclusion was that that *NR1I2 *rs3814055 was the most significant variant among the 12 selected populations, followed by *VDR* rs1540339. Using genetic structure analysis and Fst values, we also concluded that the genetic backgrounds of the Blang were similar to CHB.

Pregnane X, encoded by the gene *NR1I2*, belongs to the nuclear hormone receptor superfamily, whose major role is to promote the detoxification and clearance of drugs and toxic xenobiotics from the body as a transcription factor (Bertilsson et al., 1998). And some CYPs (Ding et al., 2015; Jin, Zhang, Shi et al., 2016a; Jin, Zhang, Geng et al., 2016b; Shan et al., 2016; Zhang et al., 2016) regulated by PXR/NR1I2 were associated with phase I metabolism in human. Moreover, some studies (Lown et al., 1997; Shimada, Yamazaki, Mimura, Inui, & Guengerich, 1994) illustrated that SNPs in PXR may be a main reason to the differences in drug reactions and the induction of CYP3A4. Rs3814055, localized in the 5’ untranslated region (UTR) of *NR1I2*, has already attracted the attention of many researchers, for both disease risk and pharmacogenomics impact. Numerous studies showed that the frequency of rs3814055 in the *NR1I2* gene varied according to different populations. The frequency of this variation in a Chinese Han population was 0.218 (Wang et al., 2007), 0.39 for Caucasians (Zhang et al., 2013), 0.21 for Asians (King et al., 2007), 0.50 for Europeans (King et al., 2007), 0.36 for the Dutch (Bosch et al., 2006), and 0.34 for African Americans (Thomas et al., 2007). In our previous studies, the frequency of the rs3814055 SNP variant in the Lhoba population and in the Miao population were 0.101 and 0.09 (He et al., 2015; Jin, Aikemu et al., 2015a), respectively. In our study about the Blangs, the allele T frequency of rs3814055 was 0.06 (Figures 1 and 2). In a Chinese Han Population, upregulated CYP3A4 expression was due to the frequency of rs3814055 (−25,385 T) (Zhang et al., 2001), demonstrating that it was similar to that of Lhoba and Miao. Yet it was still lower than the other populations. Additionally, another report has shown that the allele C linked to Inflammatory Bowel Diseases (IBD) in a European population (Martínez et al., 2010). However, the haplotype TCC of rs3814055/rs6784598/rs2276707 functioned as a whole in risk assessment for ulcerative colitis (UC) in Spanish population. In addition, Kurzawski M et al revealed that there were significant differences in tacrolimus concentrations between patients with different NR1I2 rs3814055: C > T genotypes (Kurzawski, Malinowski, Dziewanowski, & Drozdzik, 2017). And Zazuli et al. (2015) found that, in Indonesian patients with tuberculosis, the TT genotype of rs3814055 had a significantly greater risk of antituberculosis drug‐induced liver injury than those of CC genotype.

The SNP rs1540339 is situated in the intron region of VDR. Previous studies have demonstrated that rs1540339 was related to the susceptibility of type 1 diabetes mellitus (T1DM) (Wang et al., 2014), colorectal cancer (Wang, Li, & Zhou2008b), and so on. The other study drew the same conclusion that the variant involved in T1DM prevention (Wang, Li et al., 2008b). Jin TB et al. reported that the frequency of rs1540339 T in the Li population was higher than the allele C, indicating that the Li group had lower sensitivity to T1DM. In our study, the allele frequencies of rs1540339 C/T in the Blang were 34% and 66%, respectively. So we guess that the Blang may have lower susceptibility to T1DM.

Considering the above results, ethnicity is an important factor for the frequency distribution and the genotype of rs3814055 can be used as a marker for detecting IBD and UC. And the Blang may have a lower susceptibility to T1DM. Although rs1540339 has not been found to be relevant in the Blang, it is noteworthy in future studies. At present, there are more teams, including Jin TB et al., devoted to disease research of SNPs (Du et al., 2016; Duan et al., 2015; Hu et al., 2014; Wang et al., 2015; Yang et al., 2016), and we hope that our data will complement the pharmacogenomics database and provide some help for the development of personalized medicine.

## DISCLOSURE

The authors have no conflicts of interest to declare.
