# Supplementary material for: Genetic analysis of pharmacogenomic VIP variants in the Blang population from Yunnan Province of China
Source: Mol Genet Genomic Med. 2019 Apr 5;7(5):e574. doi: 10.1002/mgg3.574 (PMC6503013; doi:10.1002/mgg3.574)
Supplement: Supplementary file 1 [file MGG3-7-e574-s001.docx]

| Supplementary Table 1 There are 80 single nucleotide polymorphisms PCR primers. | | | | |
| --- | --- | --- | --- | --- |
| SNP_ID | Allele | Reverse primer（5'-3'）for PCR | Forward primer（5'-3'）for PCR | Primer for extension reactions |
| rs10264272 | C/T | ACGTTGGATGGGATCTAAGAAACCAAATT | ACGTTGGATGCGACTCTCTCAACAATCCAC | aTTTGTGGAGAGCACTAA |
| rs1042713 | A/G | ACGTTGGATGGAACGGCAGCGCCTTCTTG | ACGTTGGATGACCCACACCTCGTCCCTTT | gCCGGCGCATGGCTTC |
| rs1042714 | C/G/T | ACGTTGGATGGAAGCCATGCGCCGGACCA | ACGTTGGATGAGACATGACGATGCCCATGC | ACACCTCGTCCCTTT |
| rs1045642 | A/C/T | ACGTTGGATGTATGGAGACAACAGCCGGGT | ACGTTGGATGAAGGCATGTATGTTGGCCTC | ctaCTTTGCTGCCCTCAC |
| rs1051266 | A/G | ACGTTGGATGAGAAGCAGGTGCCCGTGGAA | ACGTTGGATGCGTAGAAGCAAAGGTAGCAC | AAAGGTAGCACACGAGG |
| rs1065776 | C/T | ACGTTGGATGAATGACCGAGGTGCTGTGGC | ACGTTGGATGTGCTGTTCCCCCAGGACGAA | ggaaCAGGACGAACCCGGACC |
| rs10735810 | T/C | ACGTTGGATGAAGTCTCCAGGGTCAGGCA | ACGTTGGATGTGGCCTGCTTGCTGTTCTTA | ccgGCTTGCTGTTCTTACAGGGA |
| rs10929302 | A/G | ACGTTGGATGTAGAGGGTAAGAGGCAGAGG | ACGTTGGATGACTCTGGGATAGACCCCAGC | CCCCAGCCCACCTGTC |
| rs1128503 | C/T | ACGTTGGATGGTTTTTTTCTCACTCGTCCTG | ACGTTGGATGTCTGCCCACTCTGCACCTT | caaTCTGCACCTTCAGGTTCAG |
| rs1138272 | C/T | ACGTTGGATGTGATACATGGTGGTGTCTGG | ACGTTGGATGTCAAAAGGCTTCAGTTGCCC | ATAGTCATCCTTGCCC |
| rs1142345 | A/C/G | ACGTTGGATGGTTGGGGAATTGACTGTCTT | ACGTTGGATGCTATGTCTCATTTACTTTTC | CTCATTTACTTTTCTGTAAGTAGA |
| rs11568820 | A/G | ACGTTGGATGCATCTTTTGTATCAGGAAC | ACGTTGGATGAACTGCAACCCATAATAAG | ACCCATAATAAGAAATAAGTTTTTA |
| rs1229984 | A/G | ACGTTGGATGCTGAATCTGAACAGCTTCTC | ACGTTGGATGTTGCCACTAACCACGTGGTC | cctcACCACGTGGTCATCTGTG |
| rs12659 | C/T | ACGTTGGATGACTGAGTCCCCACAGGCCA | ACGTTGGATGCTTCGGAGCTGGAGCGCAT | TGGAGCGCATGAATCC |
| rs12720441 | C/G/T | ACGTTGGATGTCGCCCCGCAGGATCTCGAT | ACGTTGGATGCACACTGGTGCATGCTGGG | CCGCCCTGTACTTCATCTCC |
| rs12721634 | C/T | ACGTTGGATGACTCACAGATAGAGGAGCAC | ACGTTGGATGTGATGGCTCTCATCCCAGAC | ccacGGAAACCTGGCTTCTCC |
| rs1540339 | A/G | ACGTTGGATGACACACATTCTCAGTGGGC | ACGTTGGATGGTGCTGAGCACACCTTGTTG | TGTTGGTGCCCACCCTAA |
| rs1544410 | A/G | ACGTTGGATGTAGATAAGCAGGGTTCCTGG | ACGTTGGATGAGCCCAGTTCACGCAAGAG | GCCTGAGTATTGGGAATG |
| rs16947 | A/G | ACGTTGGATGTCACCATCCCGGCAGAGAA | ACGTTGGATGCCCTGAGAGCAGCTTCAATG | gaatCTTCAATGATGAGAACCTG |
| rs1695 | A/G | ACGTTGGATGTGGTGCAGATGCTCACATAG | ACGTTGGATGATGGTGAATGACGGCGTGGA | cagtACCTCCGCTGCAAATAC |
| rs17238540 | G/T | ACGTTGGATGATGTATCACTCACCTCTAT | ACGTTGGATGTTGGACACAATGGATTAGGC | gtagTGGATTAGGCTGATATGAC |
| rs17244841 | A/T | ACGTTGGATGGGGAAACAAAGTGAGACTAC | ACGTTGGATGCAGGTATTCAAGATACAAAG | AAGTATGATTGTAATATAAAGGATTT |
| rs1799853 | C/T | ACGTTGGATGACCCACCCTTGGTTTTTCTC | ACGTTGGATGCTGCGGAATTTTGGGATGGG | agGAGGAGCATTGAGGAC |
| rs1800460 | A/G | ACGTTGGATGACTTACCATTTGCGATCACC | ACGTTGGATGGCAAATTTGACATGATTTGGG | CATGATTTGGGATAGAGGA |
| rs1800462 | C/G | ACGTTGGATGCCAAGTTCACTGATTTCCAC | ACGTTGGATGGTAAATGTATGATTTTATGC | AATGTATGATTTTATGCAGGTTT |
| rs1800497 | C/T | ACGTTGGATGTGTGCAGCTCACTCCATCCT | ACGTTGGATGTCAAGGGCAACACAGCCATC | aaagCCTCAAAGTGCTGGTC |
| rs1800566 | C/T | ACGTTGGATGGATTTGAATTCGGGCGTCTG | ACGTTGGATGATTTCTGTGGCTTCCAAGTC | agggGTGGCTTCCAAGTCTTAGAA |
| rs1800888 | C/T | ACGTTGGATGTACCAGTGCATCTGAATGGG | ACGTTGGATGTAAGGCCCGGGTGATCATTC | gggaGATTGTGTCAGGCCTTA |
| rs1801030 | A/G | ACGTTGGATGAAGATCCTGGAGTTTGTGGG | ACGTTGGATGATCTCCTTGAACGACGTGTG | ACGTGTGCTGAACCA |
| rs1801131 | A/C | ACGTTGGATGTCTCCCGAGAGGTAAAGAAC | ACGTTGGATGAGGAGCTGCTGAAGATGTGG | ccccaGAGCTGACCAGTGAAG |
| rs1801133 | C/T | ACGTTGGATGACCTGAAGCACTTGAAGGAG | ACGTTGGATGGAAAAGCTGCGTGATGATG | gGCGTGATGATGAAATCG |
| rs1801253 | C/G | ACGTTGGATGAGCCCTGCGCGCGCAGCAGA | ACGTTGGATGCCTTCAACCCCATCATCTAC | CGCAAGGCCTTCCAG |
| rs1801272 | A/T | ACGTTGGATGTCCCCTGCTCACCGCCAGT | ACGTTGGATGCATCGAGGAGCGCATCCAG | ccctGCTTCCTCATCGACGCCC |
| rs1805124 | A/G | ACGTTGGATGATGATGAAAACAGCACAGCG | ACGTTGGATGGGGCCAGGGCACCAGCAGT | aaagtGCACCAGCAGTGATGTG |
| rs2032582 | A/G/T | ACGTTGGATGGAAAATGTTGTCTGGACAAGC | ACGTTGGATGCATATTTAGTTTGACTCACC | ccagTTGACTCACCTTCCCAG |
| rs2046934 | C/T | ACGTTGGATGCAATTTCACTTATCTCTGG | ACGTTGGATGTATGGCATCTACATCTTGGG | ggaggTCTTGGGAATTTGAAATGAC |
| rs2066702 | C/T | ACGTTGGATGCTCTATTGCCTCAAAACGTC | ACGTTGGATGGCATGTGGGTTGTCTAAATG | gctaTTCTTTCCTATTGCAGTATC |
| rs2066853 | A/G | ACGTTGGATGCCTAGGCATTGATTTTGAAG | ACGTTGGATGCACCAGAAAAATCATTTCTGA | TTTTTCATTCTGCATGTGT |
| rs2228570 | A/C/G/T | ACGTTGGATGTGGCCTGCTTGCTGTTCTTA | ACGTTGGATGAAGTCTCCAGGGTCAGGCA | cctgGCTGGCCGCCATTGCCTCC |
| rs2239185 | C/T | ACGTTGGATGACAGCAACACAATTCCAGTC | ACGTTGGATGGTGACATTTACACCCTCCTC | CCCTCCTCTGTCTTCAC |
| rs2740574 | A/G | ACGTTGGATGGAAACTCAAGTGGAGCCATT | ACGTTGGATGGAATGAGGACAGCCATAGAG | acatCCATAGAGACAAGGGCA |
| rs28371706 | A/C/T | ACGTTGGATGGCTGCTTGCCTTGGGAACG | ACGTTGGATGGCCGACCGCCCGCCTGTG | GCCCGCCTGTGCCCATCA |
| rs28371725 | A/G | ACGTTGGATGGAGCCCATCTGGGAAACAGT | ACGTTGGATGTCCCAGCAAAGTTCATGGGC | gaCCCGCCTGTACCCTT |
| rs28399433 | G/T | ACGTTGGATGTGGGATGATAGATGGTGACG | ACGTTGGATGGTAATCAGCCAAAGTCCATC | aagaaATCCCTCTTTTTCAGGCAGTA |
| rs28399444 | G/A | ACGTTGGATGACTGGAAGATTCCTAGCATC | ACGTTGGATGTCTTTGGGGACCGCTTTGAC | ccaCGCTTTGACTATAAGGACA |
| rs28399454 | A/G | ACGTTGGATGGTGATCCACGAGATCCAAAG | ACGTTGGATGGTGTCCTTTTTGACTCTGCG | ggatGGCCAAACTCATGGGGATCA |
| rs28399499 | C/T | ACGTTGGATGTCTGTACAGAGAGAGTCTAC | ACGTTGGATGTTGGCTCGGTCATGAAGCTC | cGCCAATCACCTGTTCA |
| rs34489327 | - | ACGTTGGATGTGATGTAGAGTGTGGTTATG | ACGTTGGATGGGACGAATGCAGAACACTTC | TATTATAGCAACATATAAAACAACTAT |
| rs36210421 | G/T | ACGTTGGATGACGTGGAGAGCAGGCTGGAT | ACGTTGGATGCACCTGCACTCCCTCACCT | CACCTGTTGAGCTGG |
| rs3745274 | A/G/T | ACGTTGGATGTTCTTCCTAGGGGCCCTCAT | ACGTTGGATGCAAAGACGATGGAGCAGATG | GTTGGCGGTAATGGA |
| rs3760091 | C/G/T | ACGTTGGATGCCTAGGGTCTGGGATTATAG | ACGTTGGATGCAAAACTCTGATGACTCAGC | ATGACTCAGCAAAAGCA |
| rs3807375 | A/G | ACGTTGGATGGGCAATGAGAGAGAACGTGT | ACGTTGGATGTCCTGAAACTCCTCCCTTAG | actaCCTTAGAGAACTTCTGCGTTTAGA |
| rs3814055 | C/T | ACGTTGGATGGAGACCACGATTGAGCAAAC | ACGTTGGATGTCACCTGAAGACAACTGTGG | cccgtTCATTTTTTGGCAATCCCAGGTT |
| rs3846662 | C/T | ACGTTGGATGCCTTAAAACTCTTCTCATTG | ACGTTGGATGGCAATAGGTGTAAGTTGGC | gatgTGCCAGTTTAAAAATACATCATA |
| rs3918290 | A/C/G | ACGTTGGATGTCACTGAACTAAAGGCTGAC | ACGTTGGATGCACCAACTTATGCCAATTCTC | TTGTTTTAGATGTTAAATCACACTTA |
| rs4124874 | A/C | ACGTTGGATGGTCATAGTAAGCTGGCCAAG | ACGTTGGATGTCCCCAAACTTCCTTTGATG | ccccTGTTCTCAAATTGCTTTGTTCA |
| rs4148323 | A/G | ACGTTGGATGGCACAGGGTACGTCTTCAAG | ACGTTGGATGAGTTGTCCTAGCACCTGACG | ccccaCTCGTTGTACATCAGAGAC |
| rs4149056 | C/T | ACGTTGGATGGAATCTGGGTCATACATGTG | ACGTTGGATGTATGGGAGTCTCCCCTATTC | tcaACGAAGCATATTACCCATGAAC |
| rs4680 | A/G | ACGTTGGATGTTTTCCAGGTCTGACAACGG | ACGTTGGATGACCCAGCGGATGGTGGATTT | ggggaGTGGATTTCGCTGGC |
| rs4986893 | A/G | ACGTTGGATGAACATCAGGATTGTAAGCAC | ACGTTGGATGGACTGTAAGTGGTTTCTCAG | tACTTGGCCTTACCTGGAT |
| rs4986909 | C/G/T | ACGTTGGATGAGGGAGGGCTCCCTTCCCA | ACGTTGGATGCGTGACCCAAAGTACTGGAC | cttGACAGAGCCTGAGAAGTTCCTCC |
| rs4986910 | C/T | ACGTTGGATGTGAAGGACTCTGATTAGAGC | ACGTTGGATGTTGGAAGTGGACCCAGAAAC | gAGAAACTGCATTGGCA |
| rs4986913 | C/T | ACGTTGGATGAGAAAATTGACTAACCTGTG | ACGTTGGATGTGCTCTAATCAGAGTCCTTC | TTCAGAACTTCTCCTTCAAA |
| rs5030656 | -/AAG | ACGTTGGATGACTGAGGCCTTCCTGGCAGA | ACGTTGGATGACGCTCAACCCACCACCCTT | GTGGCAGCCACTCTCACCT |
| rs5629 | A/C | ACGTTGGATGTTCATCACCCGCGAGGTTGT | ACGTTGGATGTCACCACGTCGCAGGTTGA | ctccCCACGTCGCAGGTTGAATTCTC |
| rs59421388 | C/T | ACGTTGGATGATTGTGGGGACGCATGTCTG | ACGTTGGATGTGGTCACCCATCTCTGGTC | ataTCTGGTCGCCGCACCTGCCCTATCA |
| rs6151031 | -/CTGGTGAG  GAGAGAACC | ACGTTGGATGAAGCCTTGTCCTGAAGACAC | ACGTTGGATGAGAGTCTAAAAGGGCACTTG | gggcCTTGTTCTCAGTAGAAGCTGG |
| rs61736512 | C/T | ACGTTGGATGACTGCTCCAGCGACTTCTTG | ACGTTGGATGTATGGGCCCGCGTGGCGCGA | ctCAGAGGCGCTTCTCC |
| rs6277 | C/T | ACGTTGGATGATTCTTCTCTGGTTTGGCGG | ACGTTGGATGACCACCAGCTGACTCTCCC | TCTCCACAGCACTCC |
| rs6791924 | A/G | ACGTTGGATGTCCCTGGCAGCCATCGAGAA | ACGTTGGATGCTCTCCTGCAAGGTGGTTG | atagCCTGCAAGGTGGTTGAGCCGC |
| rs689466 | A/G | ACGTTGGATGATTAGATGGAAGGGAGATT | ACGTTGGATGCCTGAGCACTACCCATGATA | CAAAAGCAAAGATGAAATTCCA |
| rs701265 | A/G | ACGTTGGATGCTCCTCTGAGGAGAAAATCG | ACGTTGGATGCATGGAAAGGGATGTAAGAC | tagaAAAAACAGTCAGTACAATGAT |
| rs7294 | A/G | ACGTTGGATGTAGATTACCCCCTCCTCCTG | ACGTTGGATGAAAAAAGAGCGAGCGTGTGG | TGGTCCATTGTCATGTG |
| rs731236 | C/T | ACGTTGGATGTTCTTCTCTATCCCCGTGCC | ACGTTGGATGTTGGACAGGCGGTCCTGGAT | cccctGTCCTGGATGGCCTC |
| rs7626962 | A/G/T | ACGTTGGATGAGTCGGCCTGAGATGCACTG | ACGTTGGATGGGATTCCAGGACCTGGAGC | TGTCAGCGACTGCCT |
| rs7975232 | A/C | ACGTTGGATGTAGAGAAGAAGGCACAGGAG | ACGTTGGATGTTGAGTGTCTGTGTGGGTGG | GGATTGAGCAGTGAGG |
| rs975833 | C/G | ACGTTGGATGATATATTAAGTCCAGTTCC | ACGTTGGATGTTAACCAACTCACAGATTGC | ATTATGTGATGATGAAATTGTATG |
| rs9923231 | A/C/G/T | ACGTTGGATGGTCAAGCAAGAGAAGACCTG | ACGTTGGATGATGCTAGGATTATAGGCGTG | caggaGTGAGCCACCGCACC |
| rs9934438 | A/G | ACGTTGGATGTTCCCCGACCTCCCATCCTA | ACGTTGGATGACATGGAATCCTGACGTGGC | GCCAGGAGATCATCGAC |
